# OnabotulinumtoxinA Urethral Sphincter Injection as Treatment for Non-neurogenic Voiding Dysfunction – A Randomized, Double-Blind, Placebo-Controlled Study

**DOI:** 10.1038/srep38905

**Published:** 2016-12-13

**Authors:** Yuan-Hong Jiang, Chung-Cheng Wang, Hann-Chorng Kuo

**Affiliations:** 1 Department of Urology, Buddhist Tzu Chi General Hospital and Tzu Chi University, Hualien, Taiwan; 2 Department of Urology, En Chu Kong Hospital, New Taipei city, Taiwan; 3 Department of Biomedical Engineering, Chung-Yuan Christian University, Taiwan

## Abstract

Non-neurogenic voiding dysfunction including dysfunctional voiding and detrusor underactivity caused by a spastic or non-relaxing external urethral sphincter can theoretically be treated by injections of botulinum A toxin into the external urethral sphincter. This randomized, double-blind, placebo-controlled trial was designed to determine the clinical efficacy of onabotulinumtoxinA urethral sphincter injections in patients with dysfunctional voiding or detrusor underactivity. Patients with medically refractory dysfunctional voiding (n = 31) or detrusor underactivity (n = 31) were randomly allocated in a 2:1 ratio to receive either onabotulinumtoxinA (100 U) (n = 38) or placebo (normal saline) (n = 24). There were no significant differences in subjective or objective parameters between patients who received onabotulinumtoxinA and those who received saline injection therapy, and the overall success rate was 43.5% (reduction in Patient perception of Bladder Condition by ≥2: onabotulinumtoxinA 36.8% vs placebo 54.2%, p = 0.114). The results were similar between the dysfunctional voiding and detrusor underactivity subgroups; however, a significant reduction in detrusor voiding pressure was only observed in dysfunctional voiding patients who received onabotulinumtoxinA. Repeat urethral sphincter onabotulinumtoxinA injections offered greater therapeutic effects in both dysfunctional voiding and detrusor underactivity patients. For patients with non-neurogenic voiding dysfunction, the success rate of onabotulinumtoxinA urethral sphincter injection was not superior to placebo.

Non-neurogenic voiding dysfunction presents therapeutic challenges to urologists because of the lack of consensus regarding diagnosis and definition as well as its broad range of causes, including bladder outlet obstruction, dysfunctional voiding, detrusor underactivity and detrusor overactivity with impaired detrusor contractility^1^,^2^,^3^,^4^. A spastic or non-relaxing external urethral sphincter is thought to be a possible cause of dysfunctional voiding and detrusor underactivity, resulting in voiding symptoms, slow or fractionated urinary flow, large post-void residual urine, and sometimes deterioration of upper urinary tract function.

Botulinum toxin A, a potent neurotoxin, can inhibit the release of neurotransmitters from efferent nerve terminals at neuromuscular junctions, thereby paralyzing muscle^5^. Botulinum toxin A injection into the urethral; sphincter is now widely applied as treatment for various types of lower urinary tract diseases, including neurogenic and non-neurogenic voiding dysfunction^6^. In 1988, Dykstra *et al*. reported the first application of onabotulinumtoxinA injection into external urethral sphincter to treat detrusor-sphincter dyssynergia in patients with spinal cord injuries^7^. In 1997, Steinhardt *et al*. were the first to report that injections of onabotulinumtoxinA into the urethral sphincter of a neurologically normal child with refractory dysfunctional voiding resulted in the resolution of urinary tract infections and incontinence episodes^8^. Since then, urethral sphincter onabotulinumtoxinA injection has been used to treat various types of neurogenic or non-neurogenic voiding dysfunction, including chronic urinary retention^9^, detrusor underactivity^10^,^11^, poor relaxation of urethral sphincter^12^, and lower urinary tract symptoms in men with small prostates^13^.

For detrusor sphincter dyssynergia, urethral sphincter injection with onabotulinumtoxinA 100 U was reported to achieve an overall satisfactory result of 60.6% with significant improvement in the reduction of voiding detrusor pressure and post-void residual urine volume, and an increase in maximal urinary flow rate^14^. In 10 pediatric patients with dysfunctional voiding, urethral sphincter injection with onabotulinumtoxinA 50–100 U resulted in self voiding without catheterization, increased maximum flow rate values and lower post-void residual volume in 90% of patients^15^. Franco *et al*. reported that increasing the onabotulinumtoxinA dose to 200–300 U resulted in increased efficacy without increasing the morbidity rate^16^. Liao *et al*. found that urethral sphincter injection with 50–100 U onabotulinumtoxinA resulted in an overall success rate of 86.7% in adults with dysfunctional voiding and a success rate of 95.7% in patients with poor relaxation of the urethral sphincter^12^. By paralyzing the urethral sphincter and reducing urethral resistance, onabotulinumtoxinA injection therapy facilitates bladder emptying, improves subjective symptoms and life quality, and reduces the need for catheterization^10^,^11^. In addition, Kuo *et al*. reported that detrusor contractility recovered in approximately 50% of patients with detrusor underactivity who received urethral sphincter onabotulinumtoxinA injections, indicating that onabotulinumtoxinA has neuromodulation effects in the lower urinary tract^17^. Urethral sphincter injection with onabotulinumtoxinA was widely applied for many lower urinary tract diseases.

Although urethral sphincter onabotulinumtoxinA injection has been shown to be an effective therapy for patients with voiding dysfunction, its application for non-neurogenic voiding dysfunctions, including dysfunctional voiding and detrusor underactivity, is still off-label. Most studies on its effects have been retrospective and lacked a control arm. In addition, onabotulinumtoxinA doses and the injection techniques varied widely among different study groups. Therefore, this prospective, randomized, double-blind, placebo-controlled trial was designed to demonstrate the actual therapeutic efficacy of urethral sphincter onabotulinumtoxinA injections for the treatment of non-neurogenic voiding dysfunctions, including dysfunctional voiding and detrusor underactivity.

## Materials and Methods

### Patients and definitions in VUDS

Participants in this prospective, randomized, double-blind, placebo-controlled clinical trial comprised patients with a three-month history of medically refractory non-neurogenic voiding dysfunction, namely dysfunctional voiding and detrusor underactivity. The diagnoses of dysfunctional voiding and detrusor underactivity were established based on the results of video-urodynamic study, which were performed according to the recommendations of International Continence Society^18^. Dysfunctional voiding was diagnosed in patients who presented with an open bladder neck but narrow membranous urethra (a spinning top appearance) on real-time fluoroscopy, a poorly relaxed urethral sphincter on electromyography, and a normal-to-high voiding pressure with a low and/or intermittent urinary flow during voiding. Detrusor underactivity was diagnosed in patients with low voiding pressure and low flow rate, a post-vid residual volume >300 mL, and a low voiding efficiency (<33%) in addition to the presence of a relaxed urethral sphincter on electrometrography during voiding. Cystourethroscopy was performed to exclude anatomic bladder outlet obstruction conditions, including benign prostatic obstruction in men, bladder neck obstruction or contracture, and urethral stricture. Other exclusion criteria included active urinary tract infection, interstitial cystitis, and occult or overt neuropathy (including cerebrovascular accidents, diabetes mellitus, multiple sclerosis, Parkinson’s disease, and spinal cord injury). (Appendix).

### External urethral sphincter injection techniques and follow-up

The clinical pharmacist preparing the solution for injection allocated patients to receive either onabotulinumtoxinA (BOTOX, Allergan, Irvine, CA) (treatment group) or placebo (normal saline, control group) by permuted block randomization code in a 2:1 ratio. The patients, study nurses, and attending doctors were unaware of which agent was being injected. The rationale for this randomization was based on that therapeutic efficacy should be better than placebo and according to the request by the Institution Review Board. All patients were treated and followed up equally without any cointervention. Each vial of onabotulinumtoxinA (100 U) was diluted with 5 ml of normal saline, resulting in 20 U onabotulinumtoxinA per 1.0 ml. Male patients received 10 transurethral urethral sphincter injections of onabotulinumtoxinA solution or normal saline using a 23 gauge needle (22 Fr, Richard Wolf, and Knittlingen, Germany); each injection site received 10 U of onabotulinumtoxinA or 0.5 ml of normal saline. By a perineal route, female patients received 5 urethral sphincter injections of onabotulinumtoxinA solution or 1.0 ml of normal saline circumferentially into the urethral sphincter except at the 6 o’clock position using a 27 gauge 1 mL syringe needle. All injections were performed in the operating room under general anesthesia.

After the injection procedures, a 16 Fr urethral Foley catheter was inserted and maintained overnight in male patients but not in female patients. Patients were discharged the following day if there were no complications. An oral antibiotic agent (cephalexin 500 mg every 6 hr) was administered for 7 days. Patients were monitored in the outpatient clinic at 2 weeks and 1 month after treatment.

### Outcome Assessments

The primary end-point was the net change in Patient Perception of Bladder Condition (PPBC) score at 1 month after the initial injection, and a reduction in PPBC score ≥2 was considered a successful result. Patients were also assessed with International Prostate Symptom Score (IPSS), including IPSS-T (total), IPSS-S (storage subscore), and IPSS-V (voiding subscore), quality of life index, and videourodynamic parameters at baseline and 1 month later. Videourodynamic parameters included cystometric bladder capacity, detrusor voiding pressure, maximum flow rate, voided volume, and post-void residual volume. If patients did not satisfy with the treatment result at 1 month, repeated urethral sphincter injection with 100 U onabotulinumtoxinA was performed regardless of patient allocation. Patients receiving a second injection were assessed in the same manner used for the first treatment. All adverse events during the peri-operative and post-operative follow-up period were recorded.

There was no similar clinical trial to assess the therapeutic efficacy of onabotulinumtoxinA on voiding dysfunction by patient’s perception of bladder condition. However, based on our previous study of therapeutic results of onabotulinumtoxinA on detrusor underactivity due to radical hysterectomy^19^, an improvement of quality of life index (the score was from 0–6, similar with the PPBC used in this trial) from 4.5 ± 2.7 vs. 2.3 ± 2.3 points (p = 0.000) after treatment was noted. The effect size was estimated to be 0.5 and the desired power of the trial was 0.8, at a significant level of 0.05, a total sample size of 64 was adequate.

All experimental methods were performed in accordance with relevant guidelines and regulations. The Research Ethics Committee of the Tzu-Chi General Hospital approved the study. (TCGH IRB101-113) Each patient was informed about the study rationale and procedures and written informed consent was obtained before onabotulinumtoxinA injection procedures. This study was also registered on ClinicalTrials.gov on November 8, 2012 with an identifier: NCT01733290. There were no external funding sources for this study.

### Statistical Analysis

The efficacy and safety evaluation will be performed on per protocol dataset. The primary conclusion will be made for the primary endpoint on the per protocol population. Continuous variables are expressed as means with standard deviations and categorical data are presented as numbers and percentages (%). We used the chi-square test for categorical comparisons of data. Differences in means of continuous variables between the groups at baseline and after treatment were tested by the Wilcoxon rank sum test. A P value of <0.05 was considered to indicate statistical significance; all tests were two-tailed. All statistical analyses were performed with the statistical package SPSS for Windows (Version 16.0, SPSS, Chicago, IL).

## Results

**Figure 1:**
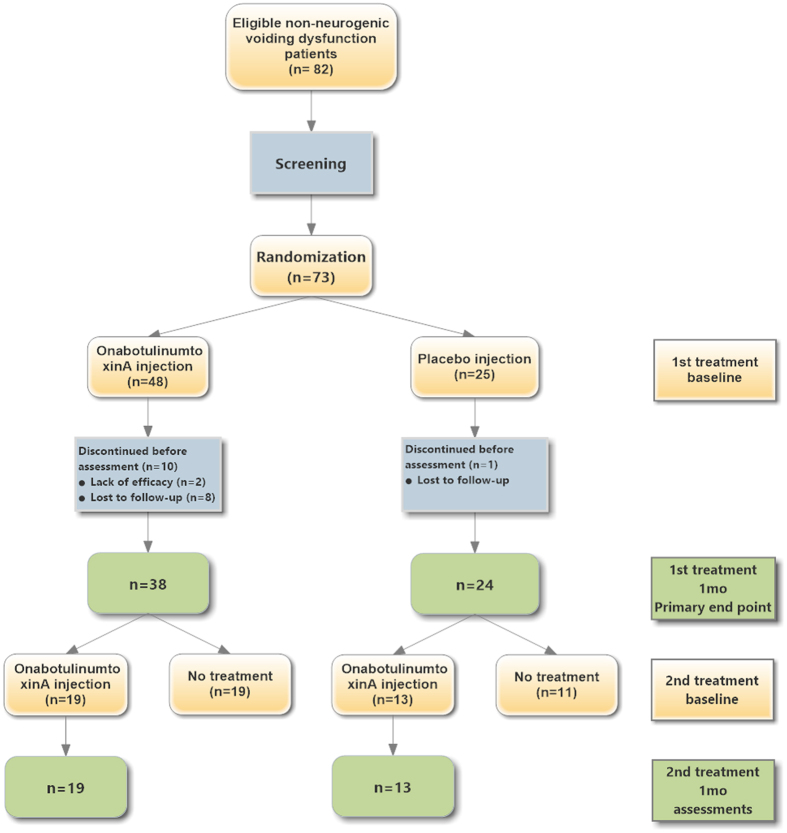
Study profile.

A total of 82 eligible patients were screened for the study and 73 were randomized for the treatment. Patients were randomized at their entry after signing informed consent form. The enrolled patients received urethral sphincter injection therapy in this study from November 2012 to April 2014, including 48 in the onabotulinumtoxinA group and 25 in the placebo group (Fig. 1). However, during the study period, 11 patients were lost to follow-up, resulting in a study population of 62 patients. Of those patients, 38 in the onabotulinumtoxinA group and 24 in placebo group completed the first injection, and 19 (50.0%) and 13 (54.2%) patients in the respective groups received the second injection (Fig.1). The participants comprised 48 women and 14 men with a mean age of 65.2 ± 15.2 years (range, 28 to 87 years). There were no significant differences in age, gender, IPSS, or videourodynamic parameters at baseline between the two groups (Table 1).

One month after the first urethral sphincter injection, the overall success rate (i.e. reduction of PPBC ≥2) was 43.5%. There was no significant difference in success rate between the onabotulinumtoxinA group (36.8%, 14 of 38) and the placebo group (54.2%, 13 of 24) (p = 0.114). In both groups, there was significant improvement in subjective clinical parameters including IPSS-V, IPSS-S, IPSS-T, quality of life index, and PPBC score. Interestingly, patients who received placebo demonstrated greater changes in IPSS-V, IPSS-T, and quality of life index than those who received onabotulinumtoxinA (Table 1). Among the videourdynamic parameters measured, maximum flow rate and voiding efficiency were significantly elevated after treatment in both groups, whereas improvements in voided volume and post-void residual volume were only observed in the onabotulinumtoxinA and placebo groups, respectively. There were no significant differences in changes in the other videourodynamic parameters between the onabotulinumtoxinA and placebo groups after the first injection. The rates of retention status decreased from 65.8% (n = 25) to 34.2% (n = 13) in the onabotulinumtoxinA group and from 58.3% (n = 14) to 12.5% (n = 3) in the placebo group.

Of the 62 treated patients, 31 had dysfunctional voiding and the others had detrusor underactivity according to detrusor voiding pressure and urethral sphincter status as measured by videourodynamic study (Table 2). Of the patients with dysfunctional voiding, 16 received onabotulinumtoxinA and 15 received placebo. Significant improvements in subjective clinical parameters including IPSS-V, IPSS-S, IPSS-T, quality of life index, and PPBC were observed in both groups after the first injection. Interestingly, there was a significantly greater reduction in IPSS-T in the placebo group than in the onabotulinumtoxinA group (p = 0.026). At one month after treatment, the success rates were 43.8% (7 of 16) in the onabotulinumtoxinA group and 66.7% (10 of 15) in the placebo group. There was no significant difference in success rate between two groups (p = 0.200). Nevertheless, significant improvements in videourodynamic parameters including detrusor pressure (from 40.3 ± 23.0 to 31.6 ± 22.3 cmH_2_O), maximum flow rate (from 6.4 ± 5.4 to 11.1 ± 10.1 mL/s) and voided volume (from 119.9 ± 82.2 to 191.0 ± 140.1 mL) were only found in the onabotulinumtoxinA group.

Of the patients with detrusor underactivity, 22 received onabotulinumtoxinA and 9 received placebo injection. Significant improvements in subjective clinical parameters including IPSS-T, quality of life index and PPBC were observed in both groups after the first injection. Interestingly, the placebo group had significantly greater reductions in IPSS-V and IPSS-T than the onabotulinumtoxinA group (p = 0.036 and 0.002, respectively). At one month after the treatment, the success rates were 31.8% (7 of 22) in the onabotulinumtoxinA group and 33.3% (3 of 9) in the placebo group. There was no significant difference in success rate between the two groups (p = 0.935). Significant improvements in maximum flow rate (onabotulinumtoxinA group from 4.5 ± 6.0 to 8.8 ± 9.1 mL/s, placebo group from 3.4 ± 3.7 to 10.6 ± 9.2 mL/s) and viding efficiency (onabotulinumtoxinA group from 20.8 ± 28.5 to 39.4 ± 36.0%, placebo group from 17.0 ± 31.5 to 61.6 ± 40.7%) were observed in both groups. Reduction in post-void residual volume, however, was significantly greater in the placebo group (from 415.0 ± 304.0 to 131 ± 169.1 mL) than in the onabotulinumtoxinA group (from 350.0 ± 174.6 to 293 ± 233.1 mL) (p = 0.046).

Of the 38 patients who received onabotulinumtoxinA, 19 (50%) underwent a second injection, including 7 patients with dysfunctional voiding and 12 with detrusor underactivity (Table 3). The other patients refusing to receive a repeat surgery included 9 satisfied with the clinical response and 10 non-responders. The success rates after the second injection increased from 28.6% to 57.1% among patients with dysfunctional voiding and from 25.0% to 50% among patients with detrusor underactivty. In patients with dysfunctional voiding, a second injection of onabotulinumtoxinA resulted in significant improvements in IPSS-S, IPSS-T, quality of life index, PPBC, and cystometric bladder capacity, which were not shown after the first injection with the exception of IPSS-T. There were no significant changes in the other videourodynamic parameters after the second onabotulinumtoxinA injection. In patients with detrusor underactivity, a second injection of onabotulinumtoxinA resulted in significant improvements in IPSS-V, IPSS-S, IPSS-T, quality of life index, PPBC, and maximum flow rate, which were not shown after the first injection, with the exception of quality of life index.

Of the 24 patients who received placebo during the first half of the study, 13 (54.2%) received an injection of onabotulinumtoxinA during the second half, including 9 patients with dysfunctional viding and 4 patients with detrusor underactivty (Table 4). The other patients refusing to receive a repeat surgery included 6 satisfied with the clinical response and 5 non-responders. In the dysfunctional voiding group, significant improvements in all subjective parameters but not in objective parameters were observed after the placebo injection during the first half of the study. In contrast, onabotulinumtoxinA injection during the second half of the study resulted in a significant reduction in detrusor pressure. No significant changes in the other parameters were noted after treatment with onabotulinumtoxinA. In patients with detrusor underactivity, significant improvements were noted in voiding efficiency after the placebo injection and in IPSS-T after the onabotulinumtoxinA injection.

Peri-operatively, there were no acute complications or adverse events after injection of onabotulinumtoxinA or placebo into the urethral sphincter, such as dysphagia, nausea, vomiting, respiratory failure, or generalized extremity paralysis. At 1 month after injection, 3 patients (4.8%) had *de novo* urgency urinary incontinence, but no one had *de novo* stress urinary incontinence. Other complications included urinary tract infection in 3 patients (4.8%), micturition pain in 2 patients (3.2%), and hematuria in 2 patients (3.2%), and all complications resolved after medical treatment.

## Discussion

This is the first prospective, randomized, double-blind, placebo- controlled trial to compare the effectiveness of onabotlinumtoxinA with that of placebo (normal saline) injections into the urethral sphincter in patients with non-neurogenic voiding dysfunction, including dysfunctional voiding and detrusor underactivity. The overall success rate was only 43.5%. Interestingly, the therapeutic effects of placebo were similar to those of onabotlinumtoxinA. Similar results were noted in the dysfunctional voiding and detrusor underactivity subgroups. Injection of either substance into the urethral sphincter might result in reduced spasticity of the urethral sphincter in patients with dysfunctional voiding and increased relaxation of the urethral shincter in patients with detrusor underactivity, regardless of the pharmacologic effects of onabotlinumtoxinA. Repeat urethral sphincter onabotlinumtoxinA injections offered greater therapeutic effects in both dysfunctional voiding and detrusor underactivity patients, indicating that a higher dose of onabotlinumtoxinA or repeat onabotlinumtoxinA delivered as a second injection is necessary for optimal pharmacologic effects in these patients.

Urinary bladder emptying requires the relaxation of the bladder neck and urethral sphincter followed by the contraction of detrusor smooth muscles, and voluntary coordinated urethral sphincter relaxation completes the voiding process^20^. Coordination between the urethral sphincter and the urinary bladder is mediated by complex neural control and reflex pathways. A poorly relaxed urethral sphincter can inhibit a forceful detrusor contraction by inhibiting the detrusor nucleus in the micturition center at the sacral cords^21^. Contraction of the urethral sphincter also activates afferents that may inhibit reflex detrusor contractions^22^. Psychogenic factors can also affect sphincter relaxation. Both a poorly relaxed urethral sphincter and an urethral sphincter with contraction during voiding not only interfere with urinary flow causing a functional bladder out obstruction but also affect the detrusor contractions contributing to bladder dysfunctions, such as detrusor underactivity. Conceptually, urethral sphincter injection with onabotlinumtoxinA might facilitate voiding by reducing urethral resistance due to its paralyzing effect and by enhancing detrusor contraction due to its potential neuromodulation effects.

Dysfunctional voiding is defined as the habitual contraction/hyperactivity of the urethral sphincter during voiding, resulting in a high voiding pressure with a low maximum flow rate and a spinning top appearance on voiding cystourethrography^23^,^24^. Urethral sphincter onabotlinumtoxinA injection has been used for pediatric and adult medically refractory dysfunctional voiding patients with a high success rate^12^,^15^,^16^. In pediatric patients, urethral sphincter onabotlinumtoxinA injection could significantly increase maximum flow rate and decrease PVR volume^25^. Franco *et al*. reported that a higher onabotlinumtoxinA dose (200–300 U) resulted in better overall outcome in post-void residual volume data compared with usual dose (50–100 U)^16^. In adult patients, Liao *et al*. reported that urethral sphincter injection with a usual onabotlinumtoxinA dose (50–100 U) resulted in a greater than 85% success rate^12^. Although high success rates were reported in all of the above-mentioned studies, all of those studies were retrospective and lacked of control groups for comparison.

In this study, urethral sphincter injection therapy with either onabotlinumtoxinA 100 U or normal saline resulted in improvement in subjective clinical symptoms and quality of life index. However, only 36.8% of patients receiving onabotlinumtoxinA injection had a successful result as measured by the PPBC improvement by >2 scales. The success rate in the placebo arm was 54.2%, suggesting that onabotlinumtoxinA urethral sphincter injection was not superior to the normal saline injection for voiding dysfunction. Interestingly, only onabotlinumtoxinA injection resulted in increased maximum flow rate and voided volume and reduced detrusor pressure, indicating that onabotlinumtoxinA has a pharmacologic effect on reduction of urethral resistance. In addition, repeat urethral sphincter onabotlinumtoxinA100 U injections resulted in improvements in subjective symptoms and in a reduction in detrusor pressure in patients who were not satisfied with the results of the first injection. That finding indicates that a higher dose or repeat urethral sphincter onabotlinumtoxinA injection might be necessary to achieve optimal pharmacologic effects in those patients.

Spastic or poorly relaxed urethral sphincter is the main pathophysiology of dysfunctional voiding. Detrusor underactivity, with a poorly relaxed or non-relaxed urethral sphincter, had a complex pathomechanism, which not only resulted from the detrusor factor itself but also involved the general medical conditions of the patients^26^. The causes of detrusor underactivity not only involves the failure of detrusor muscle activity but also failure of detrusor activation, possibly due to contraction of the urethral sphincter^22^,^27^. Therefore, the urethra is the important therapeutic target in patients with detrusor underactivity. In a study involving patients with low detrusor contractility, 48% (13 of 27) of patients who received an injection of onabotulinumtoxinA 50–100 U into the urethral sphincter showed improvement in detrusor contractility, indicating the neuromodulation effects between the urethral sphincter and bladder^17^. The overall success rate in that study was 88.9% (24 of 27) and patients in both the recovery and non-recovery groups showed significantly decreased post-void residual volume. In the present study, urethral sphincter injection therapy with either onabotulinumtoxinA 100 U or normal saline resulted in significant improvement in subjective clinical symptoms, quality of life index, and objective videourodynamic parameters including maximum flow rate and voiding efficiency but not detrusor pressure. There was no evidence of recovery in detrusor contractility, probably due to the heterogeneity of patients with detrusor underactivity and the small number of patients recruited in the study. For patients with detrusor underactivity, urethral sphincter onabltulinumtoxinA injection might result in a reduction in urethral resistance, which allowed patients to void more easily with the aid of abdominal pressure. However, if the patient is weak and cannot generate adequate abdominal pressure to void, voiding difficult and large post-void residual volume might persist. In addition, an open bladder neck is an important factor that abdominal pressure can passively overcome the urethral resistance. If patients with detrusor underactivity cannot open the bladder neck by abdominal straining, urethral sphincter onabotulinumtoxinA injection might not be successful. Furthermore, as in patients with dysfunctional voiding, repeat urethral sphincter onabtulinumtoxinA 100 U injections in some detrusor underactivty patients resulted in a reduction in subjective symptom score and an increase in maximum flow rate, indicating that a higher dose or repeat urethral sphincter onabotlinumtoxinA injection was necessary to achieve pharmacologic effects regardless of the voiding pressure.

Local injection of normal saline into the urethral sphincter resulted in significant improvement in all subjective parameters as well as in maximum flow rate and voiding efficiency. These findings indicate that the action of a local injection itself might have a therapeutic effect on the urethral sphincter activity, regardless of the pharmacologic effects of onabtulinumtoxinA. However, only urethral sphincter onabotulinumtoxinA injection in patients with dysfunctional voiding resulted in a reduction in detrusor pressure, which demonstrates the paralytic effect of onabotulinumtoxinA. Transvaginal peripheral bladder denervation for the treatment of urge incontinence was first described by Ingelman-Sundberg in 1959^28^. Other modified cystolysis procedures for the treatment of detrusor overactivity or urge incontinence have also been reported^29^. Stimulation of the urethral sphincter via solution injection might provide partial urethrolytic effects on a spastic, poorly relaxed and non-relaxed urethral sphincter, resulting in ameliorating voiding symptoms and facilitating bladder emptying.

In the present study, the success rate of onabotulinumtoxnA (36.8%) and that of placebo (54.2%) were markedly lower than the success rates reported in previous studies (range, 88.5% to 88.9%)^12^,^17^. The possible reason for this discrepancy is a stricter definition of successful result (i.e. a reduction of PPBC by ≥2). In fact, our previous study also showed only 40% of patients had an excellent result after urethral sphincter onabotulinumtoxinA injection. The major causes of failed urethral sphincter onabotulinumtoxinA injection therapy in pediatric and in adult patients with dysfunctional voiding were low detrusor contractility with low abdominal straining pressure, bladder neck obstruction, and psychological problems^12^,^25^. In addition, because of the unclear definition and pathophysiology of detrusor underactivty, eligible patients were inevitably heterogeneous. All of above conditions might affect the treatment results in the present study. Kuo *et al*. reported that in spinal cord injured patients with detrusor sphincter dyssynergia, increased continence grade and/or *de novo* urge urinary incontinence (48.5%) were the main reasons for patient dissatisfaction with urethral sphincter onabotulinumtoxinA injection therapy^14^. In the current study, there were no cases of post-procedural *de novo* stress urinary incontinence and the rate of *de novo* urge incontinence was low (4.8%), indicating that urethral sphincter onabotulinumtoxinA injection therapy for non-neurogenic voiding dysfunction without overt detrusor overactivity was a safe procedure.

The first limitation of this clinical trial was the small patient number and high withdrawal rate in the onabotulinumtoxinA group (10 of 48, 20.8% VS 1 of 25, 4% in placebo group). Second, the heterogeneous of non-neurogenic voiding dysfunction, especially for detrusor underactivity, might lead to different clinical outcome owing to different underlying pathogenesis of voidinmg dysfunction. The third limitation was the relatively short follow-up period. A longer follow-up might reveal better pharmacologic effects of onabotulinumtoxinA on the urethral sphincter and eliminate the placebo effects. The fourth limitation concerns the differences in injection techniques between male and female patients. Finally, insufficient dose of onabotulinumtoxinA administered to the urethral sphincter may be another important determinant of clinical response. Based on these limitations, further clinical trials that compare different doses of onabotulinumtoxinA and placebo in carefully selected patient groups of voiding dysfunction are needed to determine the most effective dose for specific patients with non-neurogenic voiding dysfunction.

## Conclusion

There was no difference between onabotulinumtoxinA 100 U and placebo used in urethral sphincter injection therapy for patients with non-neurogenic voiding dysfunction. However, urethral sphincter injection with either onabotulinumtoxinA 100 U or placebo could safely and effectively ameliorate clinical symptoms and improve voiding in patients with dysfunctional voiding or detrusor underactivity. Although the exact pathomechanism is unknown, local injection itself might have a therapeutic effect on the relaxation of the urethral sphincter, regardless of the pharmacologic effects of onabotulinumtoxinA. Our data also indicate that increase of onabotulinumtoxinA dose or repeat injections may render more benefits to both dysfunctional voiding and detrusor underactivity patients regardless of their voiding pressure.

## Additional Information

**How to cite this article**: Jiang, Y.-H. *et al*. OnabotulinumtoxinA Urethral Sphincter Injection as Treatment for Non-neurogenic Voiding Dysfunction – A Randomized, Double-Blind, Placebo-Controlled Study. *Sci. Rep.* **6**, 38905; doi: 10.1038/srep38905 (2016).

**Publisher's note:** Springer Nature remains neutral with regard to jurisdictional claims in published maps and institutional affiliations.

## Supplementary Material

Supplementary Appendix

## Figures and Tables

**Table 1:** Demographics and the changes of symptom scores and VUDS parameters in the onabotulinumtoxinA (BoNT-A) and placebo groups at baseline and 1 month after the first external urethral sphincter injection therapy.

		BoNT-A (N = 38)	Placebo (N = 24)	P value$	P value#
Age (yr)		64.7 ± 16.2	66.9 ± 14.2	0.562	
Gender		9 male, 29 female	5 male, 19 female	0.865	
IPSS-V	BL	15.2 ± 5.6	14.5 ± 6.7	0.468	0.002
	1 mo	12.7 ± 7.0*	6.0 ± 6.6*		
IPSS-S	BL	10.7 ± 4.0	11.0 ± 4.3	0.518	0.074
	1 mo	8.5 ± 3.8*	7.1 ± 4.2*		
IPSS-T	BL	25.8 ± 8.2	25.5 ± 8.8	0.776	0.001
	1 mo	21.2 ± 8.6*	13.1 ± 9.5*		
QoL-I	BL	4.5 ± 1.9	5.4 ± 0.9	0.024	0.014
	1 mo	3.0 ± 1.9*	2.4 ± 1.9*		
PPBC	BL	4.8 ± 1.7	5.0 ± 1.7	0.761	0.066
	1 mo	3.4 ± 2.0*	2.7 ± 2.1*		
CBC (mL)	BL	378.9 ± 154.2	397.8 ± 223.5	0.585	0.201
	1 mo	404.6 ± 182.4	360.0 ± 140.7		
Pdet (cmH2O)	BL	22.7 ± 24.7	25.3 ± 24.6	0.444	0.161
	1 mo	19.2 ± 19.6	30.5 ± 25.1		
Qmax (mL/s)	BL	5.3 ± 5.7	6.3 ± 5.1	0.344	0.558
	1 mo	9.8 ± 9.5*	9.5 ± 6.6*		
Vol. (mL)	BL	104.8 ± 112.2	102.4 ± 101.4	0.942	0.627
	1 mo	170.7 ± 140.5*	148.5 ± 144.7		
PVR (mL)	BL	295.7 ± 194.1	279.3 ± 246.9	0.965	0.141
	1 mo	251.7 ± 214.0	146.6 ± 160.5*		
VE (%)	BL	29.6 ± 28.3	34.4 ± 34.2	0.530	0.336
	1 mo	44.1 ± 35.3*	56.1 ± 36.4*		

^$^p value between the baseline data of BoNT-A and placebo groups.

^#^p value between the changes of the parameters after external urethral sphincter injection therapy in BoNT-A and placebo groups.

^*^p value <0.05 versus baseline.

BL, baseline; IPSS, International Prostate Symptom Score; IPSS-V, IPSS voiding subscore; IPSS-S: IPSS storage subscore; IPSS-T, total IPSS score; PPBC, Patient Perception of Bladder Condition score; QoL-I, quality of life index; CBC, cystometric bladder capacity; Pdet, detrusor voiding pressure; Qmax, maximal urinary flow rate; Vol, voided volume; PVR, post-void residual; VE, voiding efficiency. Data are expressed as mean ± standard deviation.

**Table 2:** The changes of symptom scores and VUDS parameters in the onabotulinumtoxinA (BoNT-A) and placebo groups at baseline and 1 month after the first external urethral sphincter injection therapy within dysfunctional voiding (DV) and detrusor underactivity (DU) patients.

		DV			DU		
		BoNT-A (N = 16)	Placebo (N = 15)	P value#	BoNT-A (N = 22)	Placebo (N = 9)	P value#
IPSS-V	BL	12.7 ± 6.6	14.1 ± 5.8		17.0 ± 3.8	15.1 ± 8.4	
	1 mo	8.3 ± 7.6*	5.7 ± 5.8*	0.089	16.0 ± 4.3	6.6 ± 8.2*	0.036
IPSS-S	BL	11.4 ± 4.3	12.1 ± 4.0		10.1 ± 3.8	9.8 ± 4.5	
	1 mo	8.5 ± 3.7*	6.3 ± 3.9*	0.089	8.5 ± 3.9	8.4 ± 4.5	0.831
IPSS-T	BL	24.1 ± 10.3	26.2 ± 7.8		27.1 ± 6.2	24.3 ± 10.7	
	1 mo	16.8 ± 10.4*	12.0 ± 8.0*	0.026	24.4 ± 5.2*	15.0 ± 11.8*	0.002
QoL-I	BL	4.4 ± 1.7	5.4 ± 1.0		4.6 ± 2.0	5.4 ± 0.9	
	1 mo	2.8 ± 2.1*	2.4 ± 2.1*	0.089	3.0 ± 1.9*	2.4 ± 1.8*	0.107
PPBC	BL	4.8 ± 1.4	5.4 ± 1.4		4.7 ± 1.9	4.4 ± 1.9	
	1 mo	3.3 ± 1.9*	2.5 ± 2.2*	0.085	3.5 ± 2.1*	3.0 ± 1.8*	0.814
CBC (mL)	BL	359.0 ± 172.6	334 ± 176		393.9 ± 141	497 ± 262.5	
	1 mo	365.0 ± 154.4	354 ± 125.7	0.860	434.7 ± 199.4	369 ± 169.2	0.042
Pdet (cmH2O)	BL	40.3 ± 23.0	35.6 ± 25.1		9.2 ± 16.4	9.1 ± 12.8	
	1 mo	31.6 ± 22.3*	32.1 ± 22.6	0.473	9.8 ± 10.0	27.9 ± 30.0	0.070
Qmax (mL/s)	BL	6.4 ± 5.4	7.9 ± 5.2		4.5 ± 6.0	3.4 ± 3.7	
	1 mo	11.1 ± 10.1*	8.9 ± 4.7	0.099	8.8 ± 9.1*	10.6 ± 9.2*	0.445
Vol. (mL)	BL	119.9 ± 82.2	136.3 ± 109		93.9 ± 130.9	45.9 ± 54.9	
	1 mo	191.0 ± 140.1*	117.5 ± 63.7	0.020	155.0 ± 142.0	200 ± 219.7	0.182
PVR (mL)	BL	225.0 ± 200.7	198 ± 168.2		350.0 ± 174.6	415 ± 304.0	
	1 mo	198.0 ± 179.1	156 ± 160.5	0.770	293.0 ± 233.1	131 ± 169.1*	0.046
VE (%)	BL	42.1 ± 27.8	44.9 ± 32.2		20.8 ± 28.5	17.0 ± 31.5	
	1 mo	50.5 ± 34.6	52.8 ± 34.6	0.969	39.4 ± 36.0*	61.6 ± 40.7*	0.094

^#^p value between the changes of the parameters after external urethral sphincter injection therapy in BoNT-A and placebo groups.

^*^p value <0.05 versus baseline.

BL, baseline; IPSS, International Prostate Symptom Score; IPSS-V, IPSS voiding subscore; IPSS-S: IPSS storage subscore; IPSS-T, total IPSS score; PPBC, Patient Perception of Bladder Condition score; QoL-I, quality of life index; CBC, cystometric bladder capacity; Pdet, detrusor voiding pressure; Qmax, maximal urinary flow rate; Vol., voided volume; PVR, post-void residual; VE, voiding efficiency. Data are expressed as mean ± standard deviation.

**Table 3:** The changes of symptom scores and VUDS parameters after the first and repeat external urethral sphincter onabotulinumtoxinA injection therapy within dysfunctional voiding (DV) and detrusor underactivity (DU) patients.

	DV (N = 7)			DU (N = 12)		
	Changes after the 1^st^ injection	Changes after repeat injection	P value#	Changes after the 1^st^ Injection	Changes after repeat injection	P value#
IPSS-V	−4.0 ± 5.5	−3.4 ± 5.4	0.848	−1.0 ± 4.0	−6.3 ± 7.0*	0.035
IPSS-S	−2.3 ± 3.9	−3.4 ± 3.3*	0.563	−2.1 ± 3.8	−4.5 ± 5.7*	0.250
IPSS-T	−6.3 ± 6.7*	−6.9 ± 3.5*	0.846	−3.0 ± 5.0	−10.7 ± 10.8*	0.051
QoL-I	−1.0 ± 2.2	−2.0 ± 1.5*	0.337	−1.8 ± 1.8*	−3.1 ± 2.2*	0.144
PPBC	−0.9 ± 2.7	−1.7 ± 1.8*	0.502	−0.9 ± 1.6	−1.8 ± 1.7*	0.202
CBC (mL)	60.6 ± 105.5	143.3 ± 143.0*	0.242	32.1 ± 72.8	77.2 ± 148.5	0.381
Pdet (cmH2O)	−8.1 ± 14.7	−13.4 ± 21.1	0.597	5.2 ± 10.7	3.8 ± 10.0	0.719
Qmax (mL/s)	1.3 ± 5.2	2.6 ± 6.0	0.676	−0.6 ± 3.8	5.4 ± 5.5*	0.008
Vol. (mL)	17.9 ± 64.7	29.7 ± 55.8	0.720	−10.3 ± 94.5	57.5 ± 150.5	0.206
PVR (mL)	29.1 ± 104.2	21.3 ± 124.7	0.900	−1.3 ± 174.4	−70.9 ± 179.3	0.367
VE (%)	−7.7 ± 25.8	−1.9 ± 31.8	0.715	−0.2 ± 17.2	19.5 ± 37.1	0.113

^#^p value between the changes of the parameters after the 1^st^ and repeat injections.

^*^p value <0.05 versus baseline (or before the injection therapy).

IPSS, International Prostate Symptom Score; IPSS-V, IPSS voiding subscore; IPSS-S: IPSS storage subscore; IPSS-T, total IPSS score; PPBC, Patient Perception of Bladder Condition score; QoL-I, quality of life index; CBC, cystometric bladder capacity; Pdet, detrusor voiding pressure; Qmax, maximal urinary flow rate; Vol., voided volume; PVR, post-void residual; VE, voiding efficiency. Data are expressed as mean ± standard deviation.

**Table 4:** The changes of symptom scores and VUDS parameters in patients receiving the 1^st^ external urethral sphincter placebo injection therapy followed by the 2^nd^ injection therapy with onabotulinumtoxinA within dysfunctional voiding (DV) and detrusor underactivity (DU) patients.

	DV (N = 9)			DU (N = 4)		
	Changes after the 1^st^ injection	Changes after the 2^nd^ injection	P value#	Changes after the 1^st^ Injection	Changes after the 2^nd^ injection	P value#
IPSS-V	−6.1 ± 4.7*	−4.5 ± 7.1	0.585	−7.0 ± 8.9	−7.8 ± 8.7	0.908
IPSS-S	−5.3 ± 4.4*	−2.9 ± 6.1	0.354	−0.0 ± 3.6	−2.3 ± 5.1	0.498
IPSS-T	−11.4 ± 7.9*	−7.4 ± 12.1	0.419	−7.0 ± 6.3	−10.0 ± 4.5*	0.468
QoL-I	−2.3 ± 2.1*	−1.1 ± 2.5	0.289	−1.75 ± 1.5	−2.5 ± 2.1	0.580
PPBC	−2.4 ± 2.1*	−1.6 ± 3.7	0.578	−1.25 ± 1.0	−2.5 ± 2.1	0.317
CBC (mL)	−10.0 ± 245.7	−29.6 ± 225.9	0.876	−133.5 ± 336.7	−88.5 ± 274.7	0.843
Pdet (cmH2O)	−3.3 ± 14.1	−8.7 ± 7.9*	0.719	33.8 ± 47.0	10.8 ± 28.8	0.436
Qmax (mL/s)	1.0 ± 5.4	1.6 ± 4.5	0.800	3.8 ± 4.3	2.5 ± 4.1	0.691
Vol. (mL)	2.2 ± 123.6	−5.0 ± 88.6	0.893	180.0 ± 260.6	69.5 ± 102.2	0.462
PVR (mL)	−54.0 ± 201.7	−107.6 ± 236.3	0.621	−400.2 ± 309.2	−251.3 ± 296.5	0.513
VE (%)	16.6 ± 27.3	15.5 ± 27.6	0.936	64.5 ± 38.9*	22.3 ± 29.1	0.133

^#^p value between the changes of the parameters after the 1^st^ and repeat injections.

^*^p value <0.05 versus baseline (or before the injection therapy).

IPSS, International Prostate Symptom Score; IPSS-V, IPSS voiding subscore; IPSS-S: IPSS storage subscore; IPSS-T, total IPSS score; PPBC, Patient Perception of Bladder Condition score; QoL-I, quality of life index; CBC, cystometric bladder capacity; Pdet, detrusor voiding pressure; Qmax, maximal urinary flow rate; Vol., voided volume; PVR, post-void residual; VE, voiding efficiency. Data are expressed as mean ± standard deviation.
