# Supplementary material for: OnabotulinumtoxinA Urethral Sphincter Injection as Treatment for Non-neurogenic Voiding Dysfunction – A Randomized, Double-Blind, Placebo-Controlled Study
Source: Sci Rep. 2016 Dec 13;6:38905. doi: 10.1038/srep38905 (PMC5153622; doi:10.1038/srep38905)
Supplement: Supplementary Appendix [file srep38905-s1.doc]

**OnabotulinumtoxinA Urethral Sphincter Injection as Treatment for Non-neurogenic Voiding Dysfunction – A Randomized, Double-Blind, Placebo-Controlled Study**

Yuan-Hong Jiang ^1^, Chung-Cheng Wang ^2-3^, Hann-Chorng Kuo ^1^*

^1^ Department of Urology, Buddhist Tzu Chi General Hospital and Tzu Chi University, Hualien, Taiwan;

^2^ Department of Urology, En Chu Kong Hospital, New Taipei city, Taiwan

^3^ Department of Biomedical Engineering, Chung-Yuan Christian University, Taiwan

*Correspondence: Dr. Hann-Chorng Kuo, Department of Urology, Buddhist Tzu Chi General Hospital, 707, Section 3, Chung Yang Road, Hualien, Taiwan

Tel: 886-3-8561825 ext. 2117

Fax: 886-3-8560794

E-mail: [hck@tzuchi.com.tw](mailto:hck@tzuchi.com.tw)

**Appendix: The inclusion and exclusion criteria of the study**

**Inclusion Criteria:**

- Adults with age of 20 years old or above.
- With the diagnosis of non-neurogenic voiding dysfunction including dysfunctional voiding and detrusor underactivity in videourodynamic study
- Free of anatomic bladder outlet obstruction on enrollment by cystourethroscopy
- Free of urinary tract infection
- Patients should have severe dysuria or urinary retention, large residual urine and have been treated with medication or other therapeutic modality for over 3 months.

**Exclusion Criteria:**

- Patients with the history of interstitial cystitis
- Patients with the history of occult or overt neuropathy (including cerebrovascular accidents, diabetes mellitus, multiple sclerosis, Parkinson’s disease, and spinal cord injury).
- Patients with severe cardiopulmonary disease and such as congestive heart failure, arrhythmia, poorly controlled hypertension, not able to receive regular follow-up.
- Patients with uncontrolled confirmed diagnosis of acute urinary tract infection.
- Patients have laboratory abnormalities at screening including: Alanine aminotransferase (ALT) > 3 x upper limit of normal range aspartate aminotransferase (AST) > 3 x upper limit of normal range.
- Patients have abnormal serum creatinine level > 2 x upper limit of normal range.
- Patients with any contraindication to be urethral catheterization during treatment.
- Female patients who is pregnant, lactating, or with child-bearing potential without contraception.
- Patients with any other serious disease considered by the investigator not suitable for general anesthesia or in the condition to enter the trial.
- Patients participated investigational drug trial within 1 month before entering this study.
